# Book review: Crossing borders, writing texts, being evaluated: cultural and disciplinary norms in academic writing by Anne Golden, Lars Anders Kulbrandstad and Lawrence Zhang

**DOI:** 10.3389/fpsyg.2023.1312287

**Published:** 2024-01-08

**Authors:** Yuehai (Mike) Xiao, Jingyi He, Li Sun

**Affiliations:** ^1^Department of English, Hunan Normal University, Changsha, China; ^2^Department of English, Wenzhou University of Technology, Wenzhou, China

**Keywords:** academic writing, cross border, English language learners, EMI, cultural differences

## Abstract

In the context of globalization, the demand for English academic writing instruction at the higher education level is experiencing exponential growth. Consequently, it is crucial to conduct research that transcends national, cultural, and disciplinary boundaries in order to address this pressing need. However, there is a scarcity of research examining the intricate and diverse nature of English academic writing instruction across a wide array of national, cultural, and disciplinary contexts, highlighting an area that calls for further investigation. In response to this gap, Anne Golden, Lars Anders Kulbrandstad, and Lawrence Jun Zhang have collaborated as editors on a comprehensive volume titled “Crossing Borders, Writing Texts, Being Evaluated: Cultural and Disciplinary Norms in Academic Writing.” This collection aims to inform educators and researchers about the challenges encountered in multilingual contexts of academic writing and propose empirically substantiated solutions and coping strategies. This article provides a summary of each chapter of the volume, highlights notable features, and offers constructive criticism by identifying areas for improvement. Overall, the authors’ adeptness in unraveling the complexity and diversity of English academic writing instruction permeates the entire book. Their utilization of empirical studies, insightful findings, practical recommendations, and consideration of diverse contexts all enable their contributions to the field of English academic writing instruction. Consequently, this book serves as an indispensable resource for researchers, educators, and graduate students who seek guidance in navigating the intricacies of teaching and learning academic writing in multilingual contexts.

## Introduction

Against the backdrop of globalization, the needs for learning and teaching English writing for academic purposes are growing exponentially in higher education (Galloway and McKinley, 2021; Xiao and Qiu, 2022). Therefore, it is imperative to conduct research on English academic writing instruction across the boundaries of nations, cultures, and disciplines. Some scholars have attempted to explore this topic. For instance, You (2016) proposed a border-crossing model for teaching writing and developing the instructors’ expertise in transliteracy in multiligual contexts. Nonetheless, limited research exists on the complexity and multiplicity of English academic writing instruction across diverse national, cultural, and disciplinary contexts, highlighting an important area for further investigation. To address this need, Anne Golden, Lars Anders Kulbrandstad, and Lawrence Jun Zhang co-edited a volume entitled “*Crossing Borders, Writing Texts, Being Evaluated: Cultural and Disciplinary Norms in Academic Writing*,” (Golden et al., 2021) aiming to inform teachers and researchers of academic writing of the challenges presented in multilingual contexts and propose solutions and coping strategies that are substantiated by empirical evidence.

The book’s premise is that academic writing is a border-crossing activity that requires understanding of cultural and disciplinary norms. The editors have gathered a collection of essays in the field to explore these norms and how they shape academic writing. The result is a rich tapestry of perspectives that can inform practitioners and researchers who are interested in the intersection of language, culture, disciplines and academic writing.

The eight chapters are written by established scholars, who had cross border experiences themselves, residing in such countries as Australia, Canada, China, New Zealand, Norway, Finland, and USA, geographically representing various areas in the world, which helps to achieve the goal of the book. The chapter authors adopted an empirical approach driven by the latest findings of theory-informed research and advancements of pedagogy. This empirical approach allowed them to provide readers with an evidence-based comprehensive and contemporary understanding of the cultural and disciplinary norms that shape academic writing and an enriching exploration of the complexity and challenges of students and teachers crossing cultural and disciplinary boundaries. As a result, most of the chapters are organized by the structure of a typical empirical research paper, covering introduction, thematic literature review, methods, findings, discussion and conclusion.

The inclusion of specific student populations in each chapter- secondary school, university, master, and doctoral students - demonstrates the book’s thoroughness in examining academic writing across different educational levels. Additionally, the consideration of international students, immigrant students, and local students in English Medium Instruction universities highlights the authors’ commitment to capturing diverse perspectives and cultural backgrounds.

The challenges and topics of academic writing being examined in this volume largely encompassed the stylistic features (technicality, density, abstraction, formality, objectivity, and rigor) of academic writing, writing assessment, language support (e.g., grammar and writing workshops), learners’ perceptions and agency,writing a summary, engaging in online discussions, writing learning journals, linguistic devices and rhetorical strategies of argumentative writing, the stakeholders’ (students, teachers, and researchers) difficulties of meeting the norms of English for academic purposes (EAP), etc.

The authors endeavor to develop connections among various cultural, linguistic and literacy traditions employed in academic writing, all of which constitute the students’ knowledge base and should be utilized to augment their academic writing skills (Wei et al., 2020). Below are the highlights of the chapter authors’ main findings and pedagogical suggestions to address the afore-mentioned challenges and our evaluative comments:

The volume comprises eight chapters, most of which are empirical studies, except for Chapter 1 (introduction) and Chapter 8 (conclusion). As an introduction to the whole book, Chapter 1 by Golden and Kulbrandstad delineates the background, rationale and underpinning theories (Contrastive and Intercultural Rhetoric and writing across disciplines) of the volume and offers a snapshot of each chapter. In Golden and Kulbrandstad’s empirical study (Chapter 2), experienced raters assessed the Norwegian language proficiency of adult learners with Spanish or Vietnamese backgrounds by reviewing two different versions of their written texts. Overall, more than half of the raters reached a consensus about categorizing each of the essays as “good,” “medium” or “weak.” Additionally, they observed a discernible pattern: After removing errors, certain texts written by Spanish-speaking test-takers received higher evaluations compared to those written by Vietnamese-speaking learners. The researchers’ alertness to cultural differences in academic writing acknowledges the challenges faced by English as an additional language (EAL) learners. This insight can help teachers and raters understand and accommodate these differences.

In Chapter 3, Rosmawati analyzed the syntactic structures used by three L2 postgraduate student writers from non-Anglophone backgrounds in Australia. She found that these participants tended to excessively use clause elaboration in their writing, suggesting a lack of understanding of the concise phrasal discourse style of academic writing. Her suggestion for explicit instruction on cross-cultural differences, especially in syntactic characteristics of English academic writing, indicates a pedagogical approach that addresses specific areas of need for EAL students.

Fang and Li (Chapter 4) investigated the difficulties faced by multilingual academic writers in American secondary schools. They analyzed writing samples from a variety of learners and gathered insights from the learners themselves regarding the challenges of cross-cultural writing. The findings unraveled that both sample texts utilized vocabulary and grammar from both academic and everyday language registers, which creates a writing style that poses rhetorical challenges and falls short of meeting the standard language expectations in a school setting. Their genre-based approach for teaching writing to teenage EAL students offers a practical approach that focuses on specific genres and promotes collaboration between English language teaching experts and subject-matter teachers.

Botelho de Magalhaes (Chapter 5) recounted the writing experiences of four Chinese PhD students at a multilingual university in New Zealand, utilizing narrative data. The findings revealed that while every person’s experiences were unique, EAL doctorate students might share some common motivations for studying abroad as well as certain challenges with overcoming language barriers and adjusting to new discourse norms. Her encouragement of EAL doctoral candidates to participate in disciplinary conversations can foster their academic identities and facilitate their integration into the academic community, which effective mentors may be able to help EAL doctoral students to navigate by overcoming the hierarchical hurdles and linguistic, cultural, and academic barriers (Xiao and Zhao, 2021a).

Leskinen (Chapter 6) explored learners’ perceptions and agency in adopting literacy practices in L2 based on a thorough qualitative investigation of students’ narratives about working with academic texts. The findings suggest that how students choose to act might be significantly influenced by their attitudes about learning, reading, and writing in a second language. Students acknowledged leveraging their bilingual resources and their expertise in using outside resources when completing projects online, and this appeared to be an important component of their strong sense of agency. Leskinen’s recognition of hierarchies of power in academic writing highlights the social dynamics involved. Acquiring academic literacy and access to the academic community are important considerations for immigrant students.

Using two written corpora, Dong (Chapter 7) analyzed the language strategies used to convey persuasiveness in the argumentative texts of L1 and L2 students. Between the two groups of writers, she discovered statistically significant variations in the employment of formulations with persuasive properties. Pathos, or the appeal to emotion, was used more frequently in the Chinese learner corpus than in the L1 corpus, although logos was used more frequently in the L1 corpus. Dong’s emphasis on the appropriate use of linguistic devices for argumentative writing in English, particularly constructing conventionalized persuasion with a confident tone, provides valuable guidance for EAL students.

In the concluding Chapter 9, Lawrence Zhang argued that EAL students should be given the chance to practice the aforementioned techniques in this book consistently in encouraging settings, particularly through the much-needed “apprenticeship” and by allowing for their repositioning in migrating geographically and interculturally.

Zhang’s call for a transformation from the problematic labels of native and non-native English speakers challenges these notions, which may derive from colonial mind-sets and promotes equality among different learner populations. It is important to reduce bias toward junior academic writers who are either EAL speakers or native English speakers (Xiao and Zhao, 2021b). We support the creation of a welcoming English-speaking environment where students are motivated to use English academic writing both inside and outside the classroom (Xiao and Zhao, 2022). It is essential to establish a multilingual and multicultural environment that fosters equality and acceptance among students, appreciating linguistic diversity as well as diverse ideas and perspectives (Xiao and Qiu, 2022).

One of the strengths of this book is its breadth of coverage in the empirical studies in the chapters, in terms of its diverse student populations, various topics in academic writing, and global geographic representations of the authors. Another merit of this book is its attention to cultural differences. Too often, academic writing is divorced from the cultural context in which it is produced, consumed, and taught. The chapters in this volume illuminate the role of cross-cultural communication in academic writing and how it shapes the teaching, learning, and research of academic writing.

Additionally, we commend the editors for assembling such a thought-provoking collection that outlines common challenges in cross-border academic writing and offers insightful pedagogical suggestions. The chapters are written in a way that is accessible to scholars from diverse disciplinary backgrounds and will be of interest to graduate students, instructors, and researchers of academic writing.

In the book, the chapter authors have effectively achieved their objective of unraveling the intricacies and variations associated with English academic writing instruction in a wide range of national, cultural, and disciplinary settings. Through conducting empirical studies, they rigorously investigated and delineated the landscape of the challenges of teaching academic writing in cross-border multilingual contexts around the world.

The findings presented in the book are not only insightful but also highly informative for researchers who seek to delve deeper into this field. By examining real-world scenarios and practical examples, the authors shed light on the complexities and nuances of teaching academic writing to students from different linguistic, cultural, and disciplinary backgrounds. These findings serve as a valuable resource for further exploration and investigation in the realm of academic writing research.

Another notable strength of the book lies in its provision of pedagogical suggestions and coping strategies that hold immense practical value for instructors of cross-border academic writing. Recognizing the unique difficulties faced by both educators and students, the authors offer tangible and effective solutions to overcome these challenges. By incorporating innovative techniques and approaches, they equip instructors with tools to enhance their teaching practices and facilitate the learning process for their students.

Furthermore, the authors successfully capture the global nature of academic writing instruction by encompassing diverse perspectives from various countries and cultures. This comprehensive approach enables readers to gain a more holistic understanding of cross-border English academic writing, as they are exposed to a range of experiences and practices from different educational systems. As a result, the book fosters a deeper appreciation for the diversity and complexity inherent in English academic writing instruction across the globe.

On the other hand, the volume is not flawless. The book offers a wide-ranging examination of the subject matter to explore multiple facets of academic writing. While the chapters do cover a diverse range of topics, readers may benefit from more effective organization of them. Perhaps the editors could group related topics together (e.g., learning, teaching, and assessing crossing border academic writing) or follow the structure of “general to specific” to organize the chapters.

Most of the studies in this book were case studies. Consequently, the research methods adopted in each empirical study mainly included interviews, narratives, corpus, and textual analysis etc. While these methods served the purposes of each study well and generated in-depth and insightful findings, more research methods could be included. To diversify the research methods and triangulate the data, a future edition of this volume may add some chapters with a wide array of data collection methods, such as questionnaires, class observations, think aloud protocols, experimental designs, etc.

Moreover, it may be helpful to add an abstract to each chapter to summarize the gist of each study, highlighting the research gaps, methods, contexts, and main findings. Furthermore, some of the chapters (e.g., Chapter 4) did not follow the structure of a typical empirical research paper, making it a bit difficult for the readers to comprehend. In Chapter 2, the appendix appeared before the references, which is not typical in a research paper either. A future edition of the book may consider addressing these concerns.

Additionally, the text could be expanded by addressing the impacts of technologies. As the world moves into the digital age, it might be helpful to explore how new communication technologies are shaping the learning, teaching, and research about cross-border academic writing. One area to explore is how new communication technologies facilitate cross-border collaboration among students, teachers, and researchers. These technologies enable real-time communication, allowing individuals from different geographical locations to engage in virtual discussions, exchange ideas, and provide feedback on academic writing. This enhances intercultural understanding and promotes a global perspective in academic writing practices (Xiao and Qiu, 2022; Xiao and Zhao, 2022).

Furthermore, the influence of new communication technologies on teaching cross- border academic writing should be examined. Online platforms, video conferencing tools, and collaborative writing software can enhance the delivery of writing instruction, providing opportunities for interactive and engaging learning experiences. Teachers can use these technologies to offer personalized feedback, conduct virtual writing workshops, and create multimedia resources that cater to diverse learners.

In terms of research, exploring how new communication technologies are shaping cross-border academic writing can shed light on emerging trends, challenges, and opportunities. Researchers can investigate the impact of digital tools on writing practices, examine the effectiveness of online writing support systems, and analyze the role of technology in fostering cross-cultural communication skills. Nonetheless, the minor caveats of this volume do not detract from its overall value.

Overall, the authors’ accomplishment in unpacking the intricacy and multiplicity of English academic writing instruction is evident throughout the book. Their use of empirical studies, insightful findings, practical recommendations, and consideration of diverse contexts are some of their contributions to the field of English academic writing instruction. As such, this book serves as an indispensable resource for researchers, educators, and graduate students seeking to navigate the complexities of teaching and learning academic writing in multilingual environments.

## Author contributions

YX: Conceptualization, Resources, Supervision, Writing – original draft. JH: Writing – original draft. LS: Writing – review & editing, Funding acquisition.
